# Uncoupling Protein-4 (UCP4) Increases ATP Supply by Interacting with Mitochondrial Complex II in Neuroblastoma Cells

**DOI:** 10.1371/journal.pone.0032810

**Published:** 2012-02-29

**Authors:** Philip Wing-Lok Ho, Jessica Wing-Man Ho, Ho-Man Tse, Danny Hon-Fai So, David Chi-Wai Yiu, Hui-Fang Liu, Koon-Ho Chan, Michelle Hiu-Wai Kung, David Boyer Ramsden, Shu-Leong Ho

**Affiliations:** 1 Division of Neurology, University Department of Medicine, University of Hong Kong, Hong Kong, Hong Kong; 2 School of Medicine and School of Biosciences, University of Birmingham, Birmingham, United Kingdom; 3 Research Centre of Heart, Brain, Hormone and Healthy Aging (HBHA), University of Hong Kong, Hong Kong, Hong Kong; The University of Hong Kong, Hong Kong

## Abstract

Mitochondrial uncoupling protein-4 (UCP4) protects against Complex I deficiency as induced by 1-methyl-4-phenylpyridinium (MPP^+^), but how UCP4 affects mitochondrial function is unclear. Here we investigated how UCP4 affects mitochondrial bioenergetics in SH-SY5Y cells. Cells stably overexpressing UCP4 exhibited higher oxygen consumption (10.1%, p<0.01), with 20% greater proton leak than vector controls (p<0.01). Increased ATP supply was observed in UCP4-overexpressing cells compared to controls (p<0.05). Although *state 4* and *state 3* respiration rates of UCP4-overexpressing and control cells were similar, Complex II activity in UCP4-overexpressing cells was 30% higher (p<0.05), associated with protein binding between UCP4 and Complex II, but not that of either Complex I or IV. Mitochondrial ADP consumption by succinate-induced respiration was 26% higher in UCP4-overexpressing cells, with 20% higher ADP:O ratio (p<0.05). ADP/ATP exchange rate was not altered by UCP4 overexpression, as shown by unchanged mitochondrial ADP uptake activity. UCP4 overexpression retained normal mitochondrial morphology *in situ*, with similar mitochondrial membrane potential compared to controls. Our findings elucidate how UCP4 overexpression increases ATP synthesis by specifically interacting with Complex II. This highlights a unique role of UCP4 as a potential regulatory target to modulate mitochondrial Complex II and ATP output in preserving existing neurons against energy crisis.

## Introduction

Mitochondrial oxidative phosphorylation generates cellular energy in the form of ATP. It involves the flow of high-energy electrons along the electron transport chain in mitochondria, from Complex I and Complex II to Complex IV to molecular oxygen, concomitantly creating a proton gradient across the inner membrane [1]. Complex V (ATP synthase) utilizes this proton gradient to drive ADP phosphorylation and generate ATP by channeling the protons back to the matrix [2]. Some electrons are diverted from this path to form reactive oxygen species (ROS) [3], [4]. Mitochondrial Complex I (NADH ubiquinone reductase) deficiency, e.g. in Parkinson's disease (PD), resulting in lower available ATP levels and ROS damage, form a vicious cycle causing further neurodegenerative damage [5], [6]. Complex I activity can be as low as 60% of normal activity in the substantia nigra of PD brain [7]. Exploring means to compensate for Complex I deficiency, presumably via Complex II, will be beneficial to reduce such pathological damages.

Uncoupling proteins (UCPs) are mitochondrial solute carriers that can regulate mitochondrial membrane potential (MMP) and ROS levels [8]–[13], preferably via their uncoupling activity that dissipates the proton gradient and reduces ROS generation during oxidative phosphorylation, so called the “mild uncoupling hypothesis” [14]. The physiological function and significance of neuronal uncoupling proteins are still unclear. However, evidence suggests that proton leak, as mainly mediated by UCPs, plays a major role in uncoupling substrate oxidation and ATP synthesis during oxidative phosphorylation [15], [16]. UCP4 is a neuronal specific UCP homologue, localized in mitochondrial inner membrane [17], [18]. Localization and functional studies suggest that UCP4 may play a role in mitochondrial function [10], [19]–[22], however, there is a lack of studies concerning the role of UCP4 in neuronal energy homeostasis, despite its distinctive distribution in the brain neurons and astrocytes [23]. UCP4 suppresses apoptosis and stimulates proliferation of adipocytes [24]. This protein can also regulate calcium homeostasis [25]; knockdown of the protein in primary hippocampal neurons resulted in calcium overload and cell death [21]. Overexpressing UCP4 reduced MMP in non-neuronal cells [17], [24], but not in neuron-derived cells under normal conditions [10]. UCP4 has been shown to be protective against oxidative stress, and to promote both glycolysis and ATP production in rat PC12 cells [21]. Recently, we found that UCP4 expression is positively regulated by the transcription factor of NF-κB via its binding site identified in the human UCP4 gene promoter region, suggesting a role for UCP4 as a novel downstream regulatory target in mitochondria responsive to the canonical NF-κB pro-survival pathway [19]. Overexpressing UCP4 in SH-SY5Y neuroblastoma cells resulted in lower oxidative stress, but also unexpectedly higher cellular ATP levels [10], which was in discrepancy with the effects of its homologues, UCP2 and UCP5 [11], [20], [26]–[29]. Our findings suggest that UCP4 possesses distinctively different properties compared with other UCP homologues. We hypothesized that the increased cellular ATP levels seen as a result of increasing expression of UCP4 was due to an increase in the efficiency of the respiratory complexes and ATP synthesis in mitochondria.

In this study, we provided evidence of how UCP4 affects mitochondrial bioenergetics. To gain this evidence we assayed the activities of the respiratory complexes, oxygen consumption, and the efficiency of ATP production. Our results indicated that UCP4 interacted with and activated Complex II activity, and consequently ATP synthesis. We suggested that UCP4-mediated proton leak may be an important factor leading to this consequence. Our results have important implications, in which mitochondrial Complex I is inhibited (e.g. in PD), regulatory effects of respiratory Complex II by UCP4 may augment ATP synthesis and thus minimize neuronal cell death due to energy deficiency.

## Results

### Overexpressing UCP4 increases total cellular ATP level

**Figure 1:**
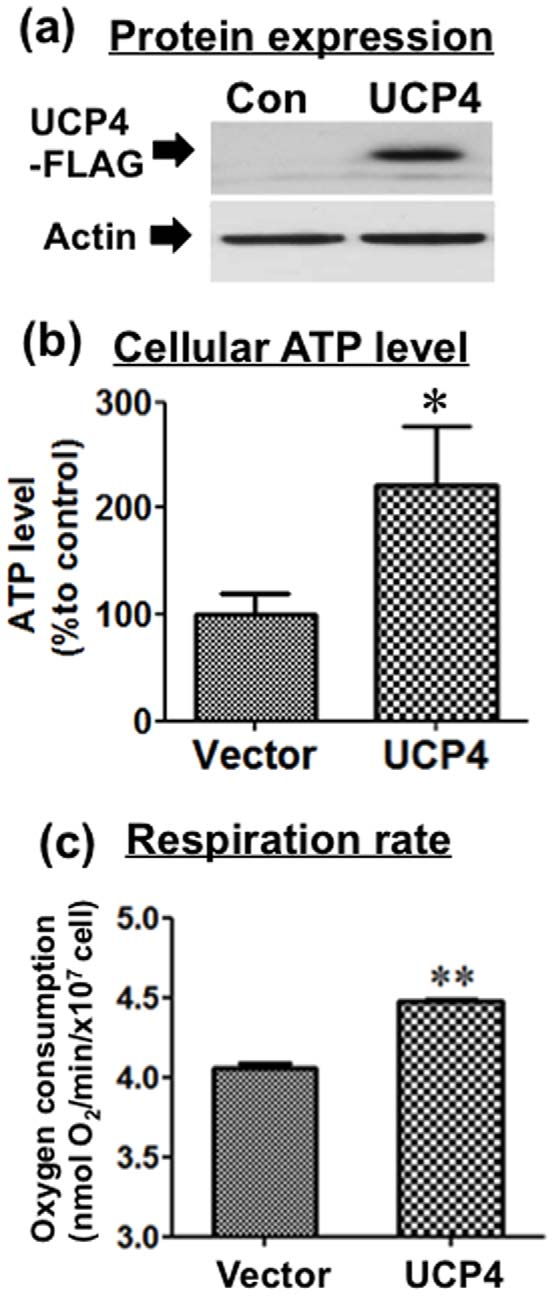
Bioenergetic characterization of SH-SY5Y cells stably overexpressing UCP4. (a) Human UCP4 protein expression level was significantly higher in UCP4-overexpressing cells than vector controls. (b) Total intracellular ATP level in UCP4-overexpressing cells was higher than vector control cells, as determined by luciferase bioluminescent assay in total cell lysates. (c) Rate of respiration in vector control and UCP4-overexpressing cells. Under normal culture condition, oxygen consumption rate of UCP4-overexpressing cells was higher than vector controls. Results are expressed as mean ± SEM based on at least three independent trials. ** represents statistical significance at p<0.01, * represents p<0.05, compared to the vector control cells.

Stable overexpression of human UCP4 in SH-SY5Y cells resulted in a significant increase in UCP4 protein (Fig. 1a). Total cellular ATP level in whole cell lysates from UCP4-overexpressing and vector control cells were determined by luciferase bioluminescent assay. Under normal, untreated culture condition, UCP4-overexpressing cells exhibited significantly higher total cellular ATP level compared with vector controls (p<0.01), as observed consistently in at least four independent clones of cells (n = 4) (Fig. 1b). The basal ATP levels in isolated mitochondria from cells with and without UCP4 overexpression were 0.227±0.01 and 0.28±0.02 µmol ATP/µg mitochondria protein, respectively.

### Neuroblastoma cells overexpressing UCP4 adopted faster respiration with higher oxygen consumption

Endogenous respiration rates of UCP4-overexpressing and vector cells were determined by measuring oxygen consumption using a Clark-type polarographic oxygen electrode under normoxic culture condition. Cells stably overexpressing UCP4 exhibited a consistently higher rate (10.1±0.4%) of oxygen consumption (4.48±0.015 nmol O_2_/min/10^7^ cell), compared with vector controls (4.05±0.029 nmol O_2_/min/10^7^ cell) (p<0.01) (n = 5) (Fig. 1c). Thus UCP4-overexpressing cells have a high respiration rate in accord with their higher total ATP level, as shown above.

### UCP4 overexpressing mitochondria produced more ATP via respiration mediated by Complex II, but not Complex I

**Figure 2:**
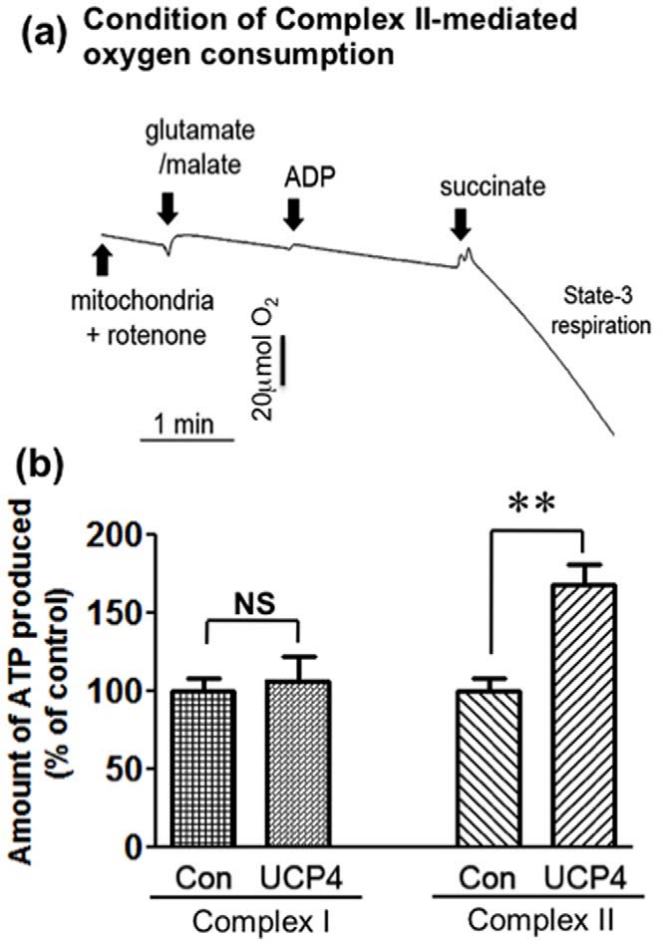
Complex II-specific oxygen consumption and ATP production of isolated mitochondria from UCP4-overexpressing and vector control cells. (a) Oxygraph showing specificity of Complex II-mediated respiration in isolated mitochondria induced by succinate. Addition of rotenone (10 µM) completely blocked Complex I activity, because addition of Complex I substrates (glutamate & malate) and ADP did not induce oxygen consumption. Subsequently, addition of Complex II substrate, succinate, induced oxygen consumption by *state 3* respiration. (b) Amount of ATP produced in isolated mitochondria from vector and UCP4-overexpressing cells utilizing either Complex I or Complex II substrates. UCP4 overexpression facilitates ATP production using Complex II substrate (succinate). There was no difference in amount of ATP produced when using Complex I substrate (glutamate/malate). Basal value of ATP produced in vector control: glutamate/malate (Complex I) = *11.42 nmol ATP/mg mitochondria protein/nmol O_2_*; succinate (Complex II): *6.68 nmol ATP/mg mitochondria protein/nmol O_2_*. Results are expressed as mean ± SEM based on at least four independent trials. ** represents statistical significance at p<0.01, compared to the vector control cells. NS: not significant.

Having demonstrated that UCP4-overexpressing cells have faster respiration with higher oxygen consumption, we investigated the role of UCP4 in enhancing the efficiency of ATP synthesis. Changes in ATP levels were determined in mitochondria isolated from UCP4-overexpressing or vector control cells in either Complex I or II-mediated respiration. Initial basal ATP levels in mitochondria were determined immediately after isolation. Addition of Complex I-substrates (glutamate & malate) and ADP induced the activities of respiratory chain Complexes I, III, IV, and V. There was no significant difference in total amount of ATP produced by mitochondria with and without UCP4 overexpression via Complex I-mediated respiration (*Vector*: 11.429±1.49 vs. *UCP4*: 11.247±0.107 nmol ATP/µg mitochondria/nmol O_2_) (n = 3) (Fig. 2b). Succinate (Complex II substrate) induced activities of Complexes II, III, IV, and V. Rotenone was included to inhibit Complex I-mediated respiration when Complex II-mediated respiration was induced. The specificity of Complex II respiration was indicated by the lack of response in oxygen consumption shown in the resultant oxygraph when Complex I substrates (glutamate & malate) and ADP were added (Fig. 2a). In contrast to activation of Complex I, the amount of ATP produced in UCP4-overexpressing mitochondria via Complex II was 67±19% higher than that in control mitochondria (*Vector*: 6.684±1.01 vs. *UCP4*: 11.566±2.14 nmol ATP/µg mitochondria/nmol O_2_; p<0.01) (n = 5) (Fig. 2b). These results demonstrated that mitochondria in which UCP4 was overexpressed produced more ATP than vector controls via Complex II, but not Complex I.

### UCP4 overexpression increases proton leak in isolated mitochondria

**Figure 3:**
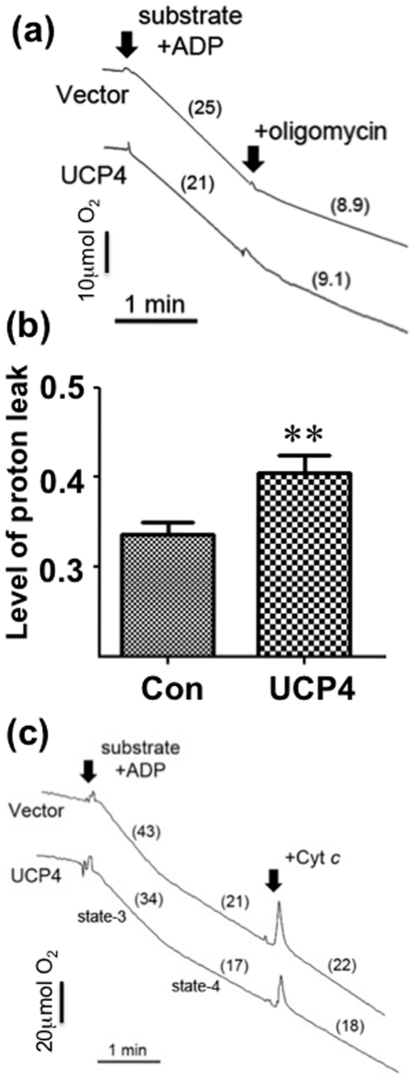
Level of proton leak and integrity of isolated mitochondria from UCP4-overexpressing and vector control cells. (a) Oxygraphs showing changes in dissolved oxygen in isolated mitochondria suspension from these cells under substrates-induced respiration. Maximum rate of oxygen consumption was recorded for 2 min after addition of substrates containing 5 mM glutamate & malate, 5 mM succinate, and 0.5 mM ADP. ATP synthase inhibitor, oligomycin (2.5 µg/ml) was then added to block ATP synthesis by Complex V. The rate of oxygen consumption with oligomycin was recorded for an additional 2 min, indicating Complex V-insensitive proton leak. Numbers in brackets represent the rate of oxygen consumption in µmol O_2_/min/mg mitochondrial protein. (b) The level of proton leak was defined as the ratio of the rate of oxygen consumption in the presence of oligomycin to the rate of substrates-stimulated oxygen consumption (*state 3* respiration). Results are expressed as mean ratios ± SEM based on at least three independent trials. ** represents statistical significance at p<0.01, compared to the vector control. (c) Representative oxygraphs reflecting integrity of the outer mitochondrial membrane of isolated mitochondria isolated from both vector control and UCP4-overexpressing cells. Exogenous cytochrome c (Cyt *c*) did not further increase mitochondrial oxygen consumption. Intact and functional mitochondria were demonstrated by *state 3* respiration as stimulated by addition of substrates (glutamate/malate/succinate) and ADP.

The degree of proton leak affects ATP production in mitochondria. Hence, the levels of proton leak were compared between UCP4-overexpressing and vector cells in isolated mitochondria. The ratio of oxygen consumption between Complex V-insensitive respiration (by addition of oligomycin) and the corresponding classical *state 3* respiration was determined. Mitochondria from UCP4-overexpressing cells had a significantly higher level (20%) of proton leak (*UCP4*: 0.405±0.019 vs. *Vector*: 0.336±0.012; p<0.01) (n = 3) (Fig. 3a–b) compared with that seen in vector controls, confirming the fact that UCP4 functions as an uncoupling protein. Because mitochondrial structural and functional integrity may be affected during the isolation process resulting in abnormal changes in oxygen consumption, the integrity of the outer mitochondrial membrane was determined in isolated mitochondria after stimulation with exogenous cytochrome *c*. There were no changes in oxygen consumption in all isolated mitochondria samples from UCP4-overexpressing or vector cells after adding cytochrome *c*, indicating that the isolated mitochondria were intact and functional (Fig. 3c).

### UCP4 overexpressing mitochondria showed higher ADP∶O ratio in Complex II-sensitive respiration, but not Complex I

**Figure 4:**
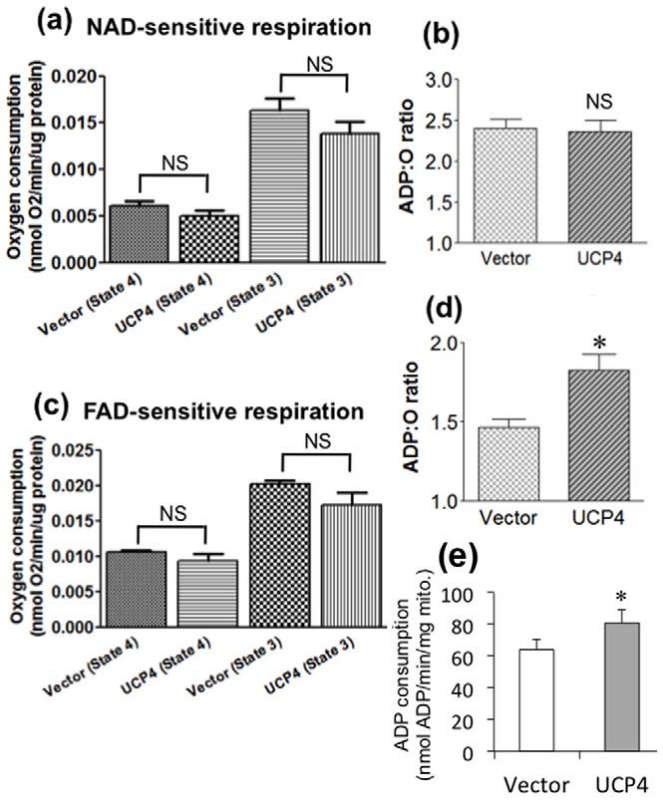
Mitochondrial oxygen consumption and ADP∶oxygen (ADP∶O) ratio of isolated mitochondria from vector control and UCP4-overexpressing cells. ADP-stimulated (*state 3*) and resting (*state 4*) respiration rates are shown for mitochondria maintained in normoxia at 25°C utilizing either (a–b) Complex I or (c–d) Complex II substrates (i.e. glutamate & malate or succinate, respectively). UCP4-overexpressing mitochondria demonstrated similar ADP∶O ratio utilizing (b) Complex I substrates, but was significantly higher when utilizing (d) Complex II substrates, as compared to vector control mitochondria. Results are expressed as mean ± SEM based on at least six independent measurements. * represents statistical significance at p<0.05, compared to the vector control. NS: not significant.

Having observed higher cellular ATP levels in UCP4-overexpressing cells associated with increased oxygen consumption, we compared the efficiency of ATP synthesis in mitochondria isolated from both UCP4-overexpressing and control cells by measuring their classical *state 4* and *state 3* respiratory rates and the ADP∶oxygen (ADP∶O) ratio using a polarographic oxygen electrode. The ADP∶O ratio was obtained from the number of moles of ATP generated per atom of oxygen consumed in the presence of either Complex I- or Complex II-specific substrates. A substrate mix of glutamate & malate was used to induce Complex I-mediated respiration. Similarly, succinate was used to induce Complex II-mediated respiration. Both classical *state 4* and *state 3* respiration rates in UCP4-overexpressing mitochondria were not significantly different from those in vector cells, although they seemed slightly lower than control cells (Table 1) (Fig. 4a, c). Interestingly, ADP∶O ratio associated with Complex II activity with succinate (Complex II-specific substrate) was 20% higher in UCP4-overexpressing mitochondria (*Vector*: 1.468±0.0494 vs. *UCP4*: 1.830±0.0986; p<0.05) (n = 5) (Fig. 4d), indicating a higher efficiency in producing ATP via Complex II. Under this condition, the ADP consumption of mitochondrial from UCP4-overexpressing cells was significantly higher than those from vector control cells (*Vector*: 64.2±6.5 vs. *UCP4*: 81.0±8.4 nmole ADP/min/mg mitochondria protein; p<0.05) (n = 4) (Fig. 4e). However, the ADP∶O ratios of both cell types for Complex I substrate (glutamate & malate) -mediated respiration (*Vector*: 2.404±0.108 vs. *UCP4*: 2.360±0.149) were similar (n = 5) (Fig. 4b). The respiratory control ratios (RCR), frequently used as an index of coupling for diagnosis of mitochondrial defects, between UCP4-overexpressing and control mitochondria induced by either Complex I- or II-specific substrates were similarly ranged within 2.5–3.0 (data not shown), being consistent with previous literatures showing functional SH-SY5Y mitochondria [30]–[32].

**Table 1:** Classical *state 4* and *state 3* respiration rate in UCP4-overexpressing and control mitochondria.

Type of isolated mitochondria	Complex I-mediated respiration (glutamate & malate)*(nmol O_2_/min/µg mito. protein)*		Complex II-mediated respiration (succinate)*(nmol O_2_/min/µg mito. protein)*	
	*state 4*	*state 3*	*state 4*	*state 3*
**Vector control**	0.006058±0.00052	0.01622±0.00131	0.01054±0.000251	0.02017±0.000601
**UCP4 overexpression**	0.00500±0.000589	0.01380±0.00124	0.009386±0.000897	0.01717±0.00182

### UCP4 overexpression increased respiratory Complex II activity, but not Complex I and IV

**Figure 5:**
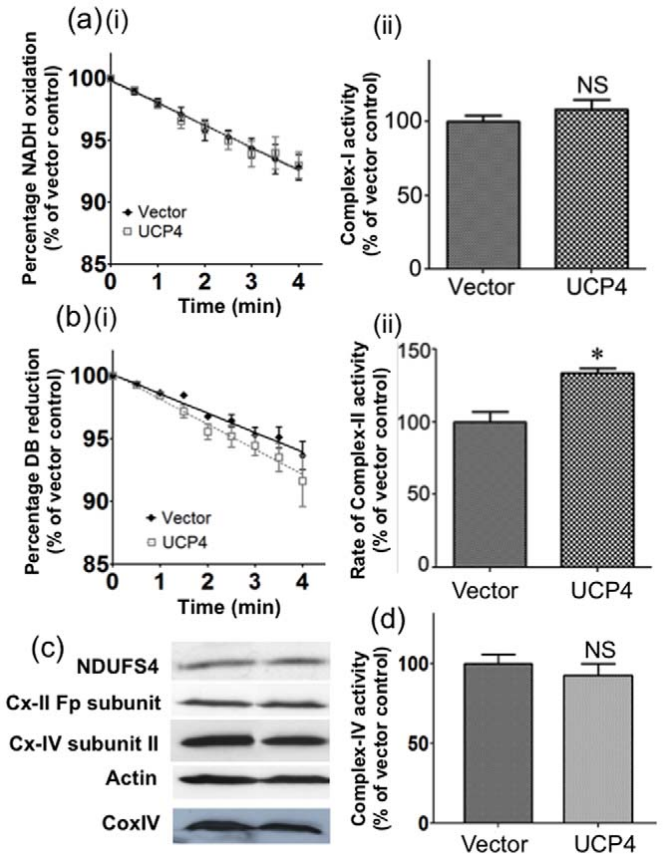
Mitochondrial Complex I, II and IV activity and expression levels in vector control and UCP4-overexpressing cells. Enzymatic activities of Complex I and II were measured in total cell lysates using spectrophotometric enzyme assay as described in *Materials and Methods*. (a)(i–ii) Complex I activity and (c) Complex I subunit, NADH dehydrogenase (ubiquinone) Fe-S protein 4 (NDUFS4) expression levels were similar in both vector control and UCP4-overexpressing cells. (b)(i–ii) Complex II in sample lysates was reactivated in 10 min pre-incubation period before assay. Complex II activity was significantly higher in UCP4-overexpressing cells, but not (c) the Complex-II Fp subunit expression level. (d) Complex IV activity and (c) Complex IV subunit II expression levels were similar in both control and UCP4-overexpressing cells. The expression level of mitochondria marker, CoxIV, reflecting mitochondrial mass, was also similar between UCP4-overexpressing and vector control cells. Results are expressed as mean ± SEM based on at least four independent trials. * represents statistical significance at p<0.05, compared to the vector control cells. NS: Not significant.

After observing an increase in ATP level and higher ADP∶O ratio under Complex II-mediated respiration in UCP4-overexpressing cells, we explored the effects of UCP4 overexpression on activities of individual respiratory complexes extracted from whole cell lysates by spectrophotometric enzyme assay. The enzymatic activity of Complex II in UCP4-overexpressing cells in normal culture was significantly higher (30%), as compared with that of vector controls (p<0.05) (n = 3), as determined by the rate of reduction of the artificial substrate, decylubiquinone (DB), in the presence of the Complex I inhibitor, rotenone (Fig. 5b). This increase in activity was not associated with significant changes in the protein level of Complex II, as shown by similar levels of the Complex II Fp subunit in the two cell types (Fig. 5c).

In contrast to the effects on Complex II, UCP4 overexpression did not alter Complex I activity as shown by similar levels of NADH oxidation rate in the two types of cells (Fig. 5a). Complex I expression was also unchanged in both UCP4-overexpressing and vector cells, as shown by similar levels of NDUFS4 (a Complex I subunit) expression (Fig. 5c). UCP4 overexpression also did not alter either Complex IV activity (n = 3) (Fig. 5d), or Complex IV subunit II protein expression (Fig. 5c). These results showed that UCP4 overexpression specifically up-regulated Complex II activity, but not those of Complex I and IV.

### UCP4 protein interacts and is co-localized with Complex II in mitochondria

**Figure 6:**
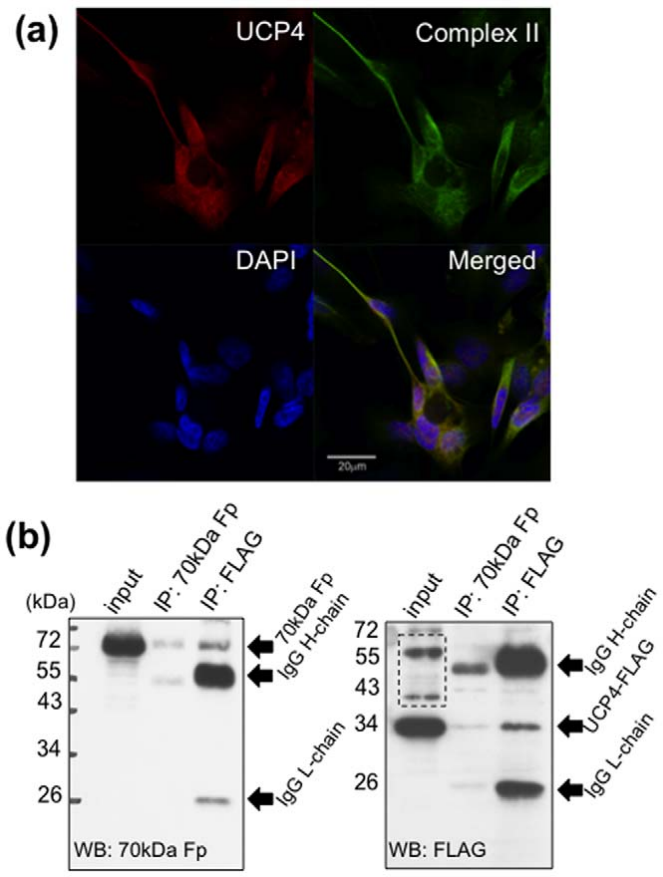
Immunocytochemistry and co-immunoprecipitation of UCP4 and mitochondrial Complex II in UCP4-overexpressing SH-SY5Y cells. (a) UCP4 (red) and a 70 kDa Complex II subunit, Fp (green), were co-localized in mitochondria. (b) Co-immunoprecipitation and western blots of recombinant UCP4 protein and Complex II subunit, Fp. Mitochondrial lysates from UCP4-overexpressing cells were immunoadsorbed (IP) with either anti-FLAG or anti-70 kDa Fp, antibody separately. The resultant antibody-protein pull-down lysates were further adsorbed by Protein-G-sepharose beads. The adsorbed proteins were cross-detected using anti-FLAG and anti-70 kDa Fp in western blots. The pull-down lysate from anti-FLAG showed a band at 70 kDa detected by anti-70 kDa Fp; and the pull-down lysate from anti-70 kDa Fp showed a band at 34 kDa detected by anti-FLAG. IgG-heavy chain and IgG-light chain were detected at ∼55 kDa and ∼25 kDa, respectively. Dashed box showed non-specific bands from crude mitochondrial lysate detected by anti-FLAG.

To elucidate protein interaction between UCP4 and Complex II, we performed co-localization study by immunocytochemistry, and co-immunoprecipitation of UCP4 and a 70 kDa Complex II subunit, Fp, using mitochondrial lysate extracted from UCP4-overexpressing cells under native condition. UCP4 protein was co-localized with Complex II as shown by double staining of antibodies against UCP4 and Complex II subunit, Fp, under confocal microscopy (Fig. 6a). For co-immunoprecipitation, in the pull-down lysate using monoclonal antibody against Complex II subunit, Fp, we detected the presence of UCP4-FLAG at 34 kDa (Fig. 6b). Simultaneously, we detected the presence of Complex II subunit, Fp, at 70 kDa in the resultant pull-down lysate using FLAG antibody (Fig. 6b). These results indicate physical binding between UCP4 and Complex II protein moiety.

### Overexpression of UCP4 in mitochondria does not affect ANT activity and expression of endogenous UCP5

**Figure 7:**
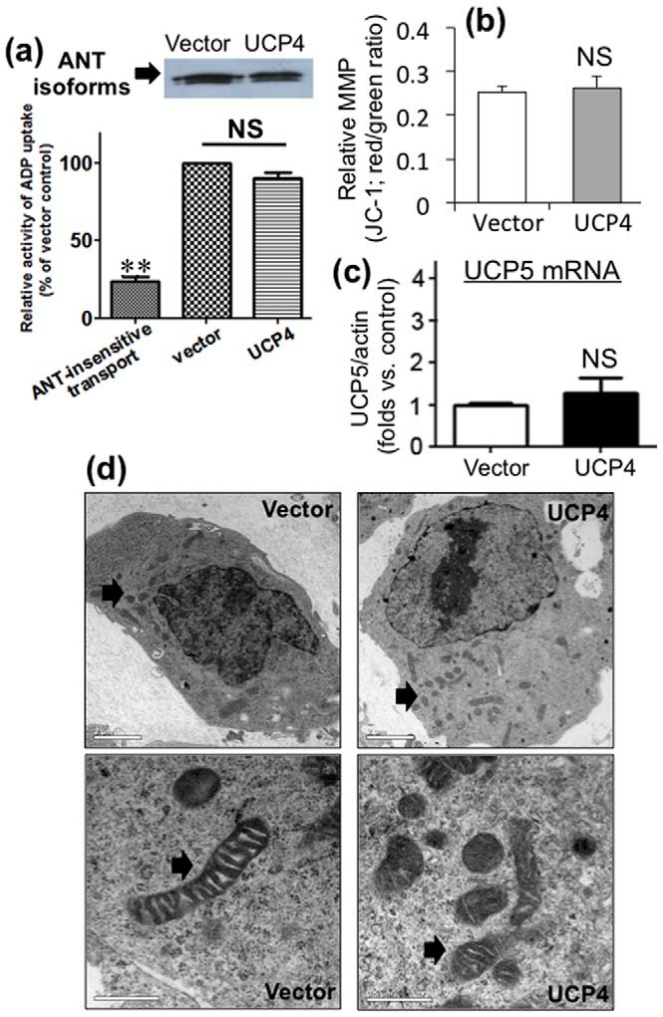
Relative changes of ADP/ATP exchange activity, ADP consumption and mitochondrial membrane potential in UCP4-overexpressing cells. (a) ANT activity in vector control and UCP4-overexpressing cells was determined using radioactive [^14^C]ADP uptake assay in isolated mitochondria. The ANT activities in both vector and UCP4-overexpressing cells were similar. Protein expression of ANT was also similar in both cell types. (b) ADP consumption in isolated mitochondria from vector control and UCP4-overexpressing cells. UCP4-overexpressing mitochondria consumed more ADP by succinate-induced *state 3* respiration. (c) Relative mitochondrial membrane potential in vector control and UCP4-overexpressing cells were similar, as determined by ratiometric (red/green ratio) measurement of JC-1 using spectrophotometry. (d) No corresponding change in UCP5 (neuronal UCP homologue of UCP4) mRNA expression in UCP4-overexpressing cells compared to vector control as shown by quantitative RT-PCR. Results are expressed as mean ± SEM based on three independent trials. ** represents statistical significance at p<0.01, * represents p<0.05, compared to the vector control cells. NS: not significant. (e) Morphology, intracellular distribution, and ultrastructure of mitochondria in vector control and UCP4-overexpressing SH-SY5Y cells were evaluated by transmission electron microscopy (TEM) at magnification of 1∶1650 and 1∶8900. The mitochondrial morphology, intracellular distribution and ultrastructure were similar in vector control and UCP4-overexpressing cells. (*Arrow*: mitochondria).

We observed increased intracellular ATP level in UCP4-overexpressing cells. Therefore, we investigated to see if the rate of ATP export from mitochondria was affected by UCP4 overexpression. We explored changes in ADP/ATP exchange activity, the expression of adenine nucleotide translocase (ANT), and the steady-state mRNA level of the neuronal UCP homologue (UCP5). These latter two particularly may affect MMP and ATP level, and thus may confound our results. The rate of ADP/ATP exchange was not significantly affected after UCP4 overexpression as demonstrated by similar level of radioactive ADP uptake via ANT (Fig. 7a). Level of ANT expression in UCP4-overexpressing cells was also similar, as compared with that of the vector control cells (Fig. 7a). UCP4 overexpression did not alter UCP5 mRNA levels compared to the level in vector controls cells (n = 3) (Fig. 7c), indicating that any potential confounding effects by UCP5 was minimal.

### Mitochondrial membrane potential (MMP) remained unchanged in UCP4-overexpressing neuroblastoma cells

The relative MMP in cells overexpressing UCP4 was compared with vector control cells by ratiometric measurement of intensity from red and green after staining with JC-1. Stable overexpression of UCP4 did not significantly alter the MMP under normal culture condition, compared with vector control cells (Fig. 7b).

### UCP4 overexpression does not affect mitochondria mass, distribution, and morphology

Under transmission electron microscopy (TEM), mitochondria from UCP4-overexpressing cells retained normal morphology *in situ* as shown by typical bean-shaped structures with well-defined outer and inner membranes and tightly packed cristae, similar to those in vector control cells (Fig. 7d). The subcellular distribution of mitochondria in UCP4-overexpressing cells was also similar to vector controls as shown under TEM (Fig. 7d). Moreover, UCP4 overexpression did not significantly alter total mitochondria mass as determined by the ratio between the expression levels of mitochondrial marker protein, CoxIV, and cytosolic house-keeping β-actin (Fig. 5c).

## Discussion

In this bioenergetic study of neuronal UCP4, we explored the role of this mitochondrial protein in the regulation of the activity of respiratory chain complexes and the efficiency of ATP synthesis in SH-SY5Y neuroblastoma cells. In aerobic respiration, mitochondria carry out oxidative phosphorylation to generate ATP from nutrients and oxygen via a series of respiratory chain complexes. Mitochondrial dysfunction impairs ATP production and forms a “vicious cycle” leading to further mitochondrial damage, which causes excessive generation of reactive oxygen species (ROS), and neuronal cell death [5]. Unlike other cell types, highly differentiated neurons continuously suppress glycolysis to maintain their antioxidant status [33]. This makes neurons highly sensitive and vulnerable to a shortage of energy supply when oxidative phosphorylation is impaired. This is particularly important for dopaminergic neurons in Parkinson's disease (PD), in which Complex I activity is significantly inhibited in the substantia nigra neurons [34].

UCP4, which is exclusively expressed in mitochondria, seems to be one of the major determinants in regulating mitochondria activity to maintain neuronal energy homeostasis. In this study, UCP4-overexpressing cells were shown to contain higher levels of ATP compared with the level in vector control cells, which is in accord with our previous findings [10]. We also demonstrate here that overexpression of UCP4 resulted in a greater level of proton leak from the inter-mitochondrial membrane space to the matrix, which is in accord with what one would expect following the increased expression of an uncoupling protein. Nevertheless, taken in conjunction with one another, these two findings are somewhat surprising, because one might anticipate that increasing proton leak via high expression of an uncoupling protein would reduce proton flow through Complex V and thus reduce ATP synthesis [14], [35]. Such a scenario occurs when another uncoupling protein, UCP5, is overexpressed [11]. Here we show that the high intracellular ATP content in UCP4-overexpressing cells was the result of increased Complex II activity. This was accompanied by higher oxygen consumption and a greater mitochondrial proton leak, but with no effects on Complex I. Interestingly, this increase in Complex II activity was not the result of increased expression of Complex II proteins but due to increased efficiency of this complex. The evidence for this is as follows: a) UCP4-overexpressing cells had a higher rate of oxygen consumption (Fig. 1c); b) UCP4 overexpression led to an enhanced rate of ATP synthesis (Fig. 2b); c) higher Complex II activity was shown by the *in vitro* enzymatic REDOX reaction (Fig. 5b); and d) the amount of oxygen consumed per ATP produced was lower, as shown by the increased ADP∶O ratio when Complex I was inhibited by rotenone and succinate was the only available substrate (Fig. 4d). However, for Complex I we did not observe any significant difference in the ADP∶O ratio or any change in oxygen consumed when Complex I was stimulated by glutamate & malate (Fig. 4a–b). One may argue that ATP can also be produced from ADP generated during *state 4* respiration, but there were no significant differences in *state 4* and *state 3* respiration between the two types of mitochondria (Fig. 4a,c).

Although we observed higher ATP levels in total cell lysates of UCP4-overexpressing cells compared with the levels in control cells, we did not observe any significant difference in either the activity or expression of ANT in isolated mitochondria from UCP4-overexpressing cells compared with the levels in control cells. One reason of the higher ATP level in UCP4-overexpressing cells is possibly due to lower ATP consumption because the demand to cope with stress responses is presumably lower in UCP4-overexpressing cells. This postulation is reasonable because there is growing evidence to show that UCP4 has a regulatory role in the control of oxidative stress and ATP-consuming apoptotic processes [10], [24], [36]. This overall effect of higher ATP production in mitochondria and lower consumption in other regions of the cell helps to explain why overexpressing UCP4 is protective against ATP deficiency induced by the mitochondrial toxin, MPP^+^, as previously reported [10]. Furthermore, we have excluded the possibility of increased mitochondrial proliferation per cell in increasing total cellular ATP because we did not observe any difference in levels of CoxIV∶actin expression ratio of UCP4-overexpressing cells compared with that of control cells.

Overexpressing UCP4 increases ATP level sounds contradictory to the classical “mild uncoupling hypothesis” in which UCPs dissipate mitochondrial membrane potential via proton leak and thereby decreasing ATP production [14]. This is true, but possibly conditional. In order to prepare for cellular stresses, normal cells should have reserved buffer of ATP production capacity to increase ATP supply when needed, possibly via regulation by UCPs. Partial uncoupling by UCPs causes proton leak from inter-membrane space to the matrix but not necessarily depolarizing mitochondrial membrane potential in live cells. An important property of proton leak is that its level being highest only under non-phosphorylating condition (i.e. classical *state 4* respiration) when ATP synthase (Complex V) is inhibited [37]. This extreme condition will seldom happen in live cells (or the cells should have died from energy deficiency), because normally proton leak will be indirectly switched off to more thermodynamically favorable route of proton entry via ATP synthase [37]. In fact, we did not observe any significant change in mitochondrial membrane potential after stably overexpressing UCP4. This is not surprising because changes in MMP in live cells is highly dynamic over a short period of time (usually within a second), and the presence of different compensatory mechanisms in the mitochondrial membrane, e.g. ANT activity [38], [39], may confound any observations and its interpretation. Any observable changes in MMP at one particular time point could be an artefactual uncoupling [40]. Therefore, any change in MMP in a live cell model should be interpreted as an overall effect from a cascade of biochemical processes. MMP will steadily decrease only in artificial membranous model (e.g. proteoliposomes) embedded with recombinant uncoupling proteins [41], because it is a simple, unidirectional flow of protons from one side of the membrane to the other without any confounding compensatory proteins. This is only true when protons, that are being pumped, are not being further “removed” or transported back to the other side of the membrane, e.g. in artificial liposomes [42]. In a whole cell system, the uncoupling function of UCPs is assumed to reduce ATP synthesis because of partial dissipation of proton gradient, which bypasses ATP synthase. However, this is valid only when it is assumed that proton gradient is built to a far higher level than required or reached the maximum capacity that ATP synthase can cope with in ADP phosphorylation (i.e. maximum ATP production). Under such hyperpolarized condition, oxidative phosphorylation also produces ROS when single electrons are transferred to molecular oxygen [43]. Only in such case, generation of harmful free radicals can be suppressed by effective dissipation of such hyperpolarized membrane potential by UCPs via proton leak. This is the reason why uncoupling activity under such condition will suppress oxidative stress, presumably by sacrificing ATP production. However, cells under unstressed condition do not necessarily need to maintain its ATP production state at maximum capacity, and expression of UCPs may not necessarily means reduction of ATP production. When compared with normal control cells, we consistently observe a higher ATP level in cells which stably overexpressing UCP4. Our results indicate that UCP4 expression plays a key role in maintaining a buffer capacity for a cell to further increase its ATP production ability via specific interaction with Complex II, which strengthen the cell in energy supply against cellular stresses.

**Figure 8:**
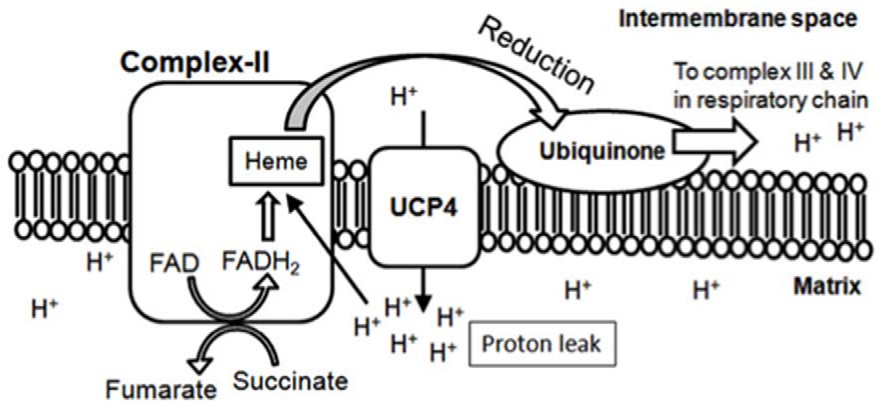
Hypothetical model showing potential role of UCP4-mediated proton leak in facilitating Complex II-mediated oxidative phosphorylation and ATP synthesis. UCP4 may span in close proximity to Complex II on the mitochondrial inner membrane to increase regional proton concentration in the matrix, and facilitate subsequent reduction of ubiquinone (UQ) via heme-group to increase Complex II activity. Proton leak via UCP4 may increase regional proton supply near Complex II, which boosts proton transport in the downstream respiratory Complex III and IV. Net effect is a facilitation of high-energy electron flow along the respiratory chain to increase ATP synthesis.

We demonstrated a unique property of UCP4 that its overexpression causes higher proton leak, and increases total cellular ATP level. Such a unique property is not observed in other UCP homologues, UCP2 and UCP5, in that they do not increase ATP production [11], [29], [44]. Based on the specific increase in Complex II activity by UCP4 overexpression, we also showed by co-immunoprecipitation that UCP4 specifically bind to mitochondrial Complex II (Fig. 6b). We believe that such interaction between UCP4 and Complex II facilitates the process of ATP synthesis. In the respiratory chain reactions, Complex II couples succinate oxidation to fumarate in the matrix with the reduction of ubiquinone (UQ) in the membrane, via the four protein subunits containing a bound flavin adenine dinucleotide (FAD) cofactor and iron-sulfur clusters. Complex II does not transport proton across the mitochondrial membrane, but reduction of ubiquinone requires four protons in which two of them are supplied by FADH_2_ carrying protons from the oxidation of succinate to fumarate; whereas the other two protons come from the proton pool in the matrix near Complex II [45]. Provided that UCP4 uniquely interact with Complex II, leakage of two protons via UCP4 may increase the regional proton concentration in close proximity to Complex II, these two additional protons carried through by UCP4 into the proton pool in matrix will potentially facilitate the downstream reduction of UQ, and consequently six additional protons will be pumped back to the inter-membrane space via downstream chain reactions in Complex III (four protons) and Complex IV (two protons). As a result, the net effect will facilitate development of proton gradient through downstream complexes and thereby driving further oxidative phosphorylation and ATP production. Such an explanation is consistent with our observation that proton leak by overexpressing UCP4 can simultaneously increase Complex II activity and ATP production. Although Complex I also extracts protons from the proton pool during reduction of UQ similar to Complex II, the activity and expression of Complex I were not significantly affected by UCP4 overexpression. The reason why UCP4 selectively activated Complex II, but not Complex I, depends on the specific protein-protein interaction between UCP4 and Complex II. When considering the fluid mosaic model of biological membrane (including mitochondrial inner membrane), one possibility may be due to specific steric interaction between these two partners (Fig. 8), which brings UCP4 protein into close proximity to supply protons to the UQ reduction site of Complex II where it hungers for protons thermodynamically. Other possibilities may be that UCP4 facilitates the dehydrogenation of succinate to fumarate to increase supply of protons to Complex II. These possibilities need to be further explored.

Examples of uncoupling proteins to regulate cellular ATP level are common in different cell types. Previous reports showed that UCP2 regulates cytosolic ATP/ADP ratio via control over glucose metabolism in pancreatic β-cells [46]. Although the expression level of UCP2 is relatively low when compared with UCP4 in neuronal cells, our previous studies have indicated functional similarities and potential compensation between UCP4 and UCP2. We found that UCP2 expression was significantly induced by mitochondrial toxin MPP^+^, and overexpressing UCP4 completely abolished the induction of UCP2 in these neuroblastoma cells [20], [47]. The role of UCP4 in neuronal energy homeostasis is further supported by another UCP4 overexpression neural cell model, which demonstrated increasing glucose uptake and shifting of the mode of ATP production from mitochondrial respiration to glycolysis, thereby maintaining cellular ATP levels [21]. Although the extent of glycolysis contributing to the overall ATP supply in neurons needs further exploration, it is not surprising that UCP4 may play a role in mitochondria to regulate ATP/ADP ratio indirectly via glycolytic pathways taken place in the cytosol. Nevertheless, this also helps to explain why we observed difference in change of ATP levels between whole cell lysates and isolated mitochondria from UCP4-overexpressing cells under Complex II-mediated oxidative phosphorylation. Furthermore, we demonstrated neuroprotective properties of UCP5 (neuronal homologue of UCP4) in attenuating ATP deficiency against mitochondrial toxicity [11]. However, we did not detect any significant change in UCP5 expression in our UCP4 overexpression model. Any potential interaction of UCP5 with the respiratory complexes, and how it affects mitochondria bioenergetic is unknown and requires further investigation.

In this bioenergetic study, we have demonstrated that overexpressing UCP4 in neuroblastoma cells under normal culture condition can increase mitochondrial ATP supply via direct protein-protein interaction with Complex II to enhance its REDOX activity. Despite that our findings in isolated mitochondria were based on exposure to non-physiological condition, i.e. at a higher oxygen and ADP concentration than the microenvironment *in vivo*, it is interesting to note the effects of overexpressing UCP4 on Complex II activity are specific, and that significant increase in ATP production induced by UCP4 was not dependent on any mitochondrial stresses. Also, one might argue for the possibility that overexpressing protein in mitochondria might cause mis-folded protein accumulation, and such impairment of mitochondrial function may confound one's observations in bioenergetic study. However, this is unlikely to happen because the isolated mitochondria in our current model have demonstrated increase in ATP production, and without undesirable oxygen consumption as shown in exogenous stimulation of cytochrome *c*. Also, the mitochondrial morphology in UCP4-overexpressing cells is normal and comparable to control cells as shown in TEM. Our data indicate that increasing expression of UCP4 in mitochondria is sufficient to sustain a higher efficiency of respiratory chain activities and ATP output under unstressed condition. To understand the yet-unknown physiological role of UCP4, evidence has emerged to show its significance [48], [49]. Recent evidence has demonstrated genetic linkages of UCP4 variants to the occurrence of various neurological disorders, e.g. multiple sclerosis [50], schizophrenia [51], [52], and leukoaraiosis [53]. In experimental animals, knocking out DJ-1 (a gene associated with an early-onset form of PD) down-regulated UCP4 expression, where the authors suggested that UCP4 (together with UCP5) may play a role to compromise calcium-induced uncoupling and increase oxidation of matrix proteins specifically in dopaminergic neurons [54]. We have recently characterized the promoter region of human UCP4 gene and identified a potent NF-κB response element in this region, where we found that TNF-α exposure in SH-SY5Y cells induced UCP4 expression via binding with NF-κB transcription factors [19]. Therefore, expression of neuronal UCP4, as a potential downstream effector, implicates significant consequences on neuronal function and determines the fate of neuronal survival implicated in various neurodegenerative pathologies. Mitochondrial dysfunction is one of the final common pathways in variety of neurological disorders, in which respiratory chain deficiency is thought to play a crucial role in determining the fate of neurons, e.g. Parkinson's disease (PD) [55]. Mitochondrial Complex I activity is reduced in substantia nigra of PD brains [56]. Current treatment of PD does not address the underlying nigrostriatal neurodegeneration. In preserving existing neurons, one such strategy involves alleviating the harmful effects of downstream pathogenic processes by targeting mitochondrial dysfunction in PD. Our current study highlights a unique role of UCP4 in regulating energy homeostasis, and may be a potential regulatory target to specifically interact with mitochondrial Complex II to increase ATP output in preserving existing neurons against energy crisis, especially when Complex I is pathologically impaired.

## Materials and Methods

### Materials

Human SH-SY5Y neuroblastoma cells were obtained from American Tissue Culture Collection (ATCC, CRL-2266). Dulbecco's Modified Eagle Medium+GlutaMAX™-1 Nutrient Mixture F-12 (DMEM-F12), fetal bovine serum (FBS), penicillin-streptomycin, Opti-MEM, Lipofectamine™ 2000, pcDNA3.1(+)expression vector and Trizol™ were from Invitrogen Life Technologies (CA, USA); adenosine 5′-diphosphate potassium salt (ADP) was from Calbiochem (CA, USA). Taqman™ EZ RT-PCR reagent kit, probes, Primer Express™ Software v.2.0, and StepOne Real-Time PCR System and StepOne Software v2.0 were from Applied Biosystems (CA, USA); Clark-type oxygen electrode in a water-jacketed microcell (model MT200; 782 2-Channel Oxygen System; Strathkelvin Instrments Ltd. Scotland); Bradford dye reagent and PVDF membrane were from Bio-Rad (CA, USA); ATPlite™ luminescence ATP detection assay system from Perkin Elmer; Complex IV Human Enzyme Activity Microplate Assay Kit was from Mitosciences (OR, USA); Polyclonal anti-actin, Monoclonal anti-NDUFS4 (1-E-4), polyclonal anti-ANT antibodies were from Santa Cruz Biotechnology (MA, USA); anti-Complex II Fp subunit and anti-Complex IV subunit II antibodies were from Mitosciences. Polyclonal anti-CoxIV was from abcam (UK). Horseradish peroxidase-conjugated secondary antibodies were from DAKO (CA, USA); ECL-plus western blotting detection system and Geneticin (G418) were from GE Healthcare Ltd. (Buckinghamshire, UK). All chemicals were from Sigma (MO, USA).

### Cell culture and treatments

Human neuroblastoma SH-SY5Y cells were cultured in DMEM-F12 supplemented with 10% FBS, 2 mM glutamine, and 100 µg/ml penicillin-streptomycin (Invitrogen) at 37°C in a humidified 5% CO_2_ atmosphere. UCP4 overexpressing SH-SY5Y cells were selected by antibiotics G418 (200 µg/ml) after stably transfected with a mammalian expression vector, pcDNA3.1(+), overexpressing human UCP4 as previously described [10]. Cells stably transfected with empty-vector were used as normal control.

### Isolation of mitochondria

The mitochondria from SH-SY5Y cells were separated based on differential centrifugations. Cells were harvested by centrifugation in a mitochondrial isolation buffer containing 10 mM Tris-MOPS, 1 mM EGTA, 0.2 M sucrose, pH 7.4, and mechanically ruptured using glass-telfon Potter Elvehjem homogenizer by 20–25 strokes. The suspension was transferred to a fresh tube and centrifuged at 650×g for 10 min. at 4 °C to obtain the mitochondria-enriched fraction. Isolated mitochondria were pelleted by further centrifugation at 7000×g for 10 min. at 4 °C. The purified mitochondria were obtained by an additional wash in isolation buffer and centrifugation at 4 °C. The mitochondria were resuspended in mitochondrial isolation buffer and kept on ice before experiments within two hours after extraction. Mitochondrial protein concentration in each sample was determined by the Bradford method and adjusted to 10 mg/ml.

### Mitochondria morphology and integrity – Electron microscopy and stimulation of cytochrome c-sensitive respiration

The ultrastructure of mitochondria in vector control and UCP4-overexpressing SH-SY5Y cells was assessed with transmission electron microscopy (TEM). For whole cell electron microscopy, cell suspension was fixed with 2.5% glutaraldehyde in phosphate buffer, pH 7.4 for 1 hour at 4°C and postfixed with 1% OsO_4_ in the same buffer. The cell pellet was pre-warmed in a 50°C water bath for 10 min. and suspended in 2% low-melting agar gel until solidified. Afterwards, samples were dehydrated, embedded in Epon resin and examined under a Philips EM208s transmission electron microscope (EMU, University of Hong Kong). The integrity of isolated mitochondria was also confirmed by tracing oxygen consumption in response to exogenous cytochrome *c* using a Clark-type oxygen electrode. Cytochrome *c* cannot penetrate an intact mitochondrial outer membrane. Therefore, the absence of an increase in oxygen consumption in isolated mitochondria in response to exogenous cytochrome *c* is a convenient qualitative confirmation of mitochondrial integrity [57].

### Rate of respiration - oxygen consumption in whole cells and isolated mitochondria

The rate of endogenous respiration was measured in either UCP4-overexpressing or vector control cell suspensions. Briefly, cells (1×10^7^) in the exponentially growing phase were trypsinized and resuspended in fresh complete medium. Changes in oxygen consumption were measured using a Clark-type oxygen electrode in a microcell, magnetically stirred at 37°C for 2 min. Oxygen consumption was recorded as nmol O_2_ consumed/min/×10^7^ cell.

Changes in oxygen consumption in isolated mitochondria from either vector control or UCP4-overexpressing cells were measured using the same oxygen electrode, magnetically stirred at 25°C. The isolated mitochondria (100 µg) were injected into the microcell using a Hamilton syringe at a final concentration of 1 mg/ml in medium (100 µl) consisting of 125 mM KCl, 1 mM EGTA, 1 mM KH_2_PO_4_, and 10 mM Tris–MOPS, pH 7.4, at 25°C. Classical *state 4* and *state 3* respiration rates were determined from the resultant oxygraphs after subsequently injecting either Complex I or II-substrates (i.e. 5 mM glutamate & malate or 5 mM succinate, respectively) and 0.1 mM ADP. The rate of respiration is defined as the rate of change of dissolved oxygen concentration in the respiration assay buffer in nmol O_2_/mg mitochondrial protein/min. *State 3* respiration was defined as the maximum rate of oxygen consumption in the presence of substrates and ADP to undergo ATP synthesis, whereas *state 4* respiration was defined as the basal oxygen consumption after ADP was depleted in the presence of substrate. To measure the oxygen consumption due to Complex II activity, 2 µM rotenone (Complex I inhibitor) was added into the mitochondrial suspension to determine the Complex I-insensitive oxygen consumption. The ADP∶oxygen ratio (ADP∶O) was determined by an ADP pulse method with definite amount of ADP (10nmoles) and Complex I and II-substrates [58]. Artificial uncoupler, CCCP, was used to fully depolarize isolated mitochondria in oxygen chamber after measurements to verify functional specificity of intact mitochondria.

### ADP consumption

The ADP consumption of isolated mitochondria from either UCP4-overexpressing or vector control cells was calculated from the oxygraph during succinate-induced *state 3* respiration via Complex II as described above. Briefly, 10nmole of ADP was added into the oxygen chamber containing 100 mg isolated mitochondria to initiate *state 3* respiration. Complete consumption of ADP was demonstrated by a distinct transition from *state 3* to *state 4* respiration as shown by a decrease of oxygen consumption. The level of ADP consumption was expressed as nmole ADP consumed per min per milligram isolated mitochondria during succinate-induced *state 3* (maximum) respiration.

### Proton leak - measurements of Complex V-insensitive oxygen consumption

The levels of proton leak in isolated mitochondria from UCP4-overexpressing and vector control cells were determined polarimetrically with a Clark-type oxygen electrode. Mitochondria were resuspended at a concentration of 1 mg/ml in medium (100 µl) consisting of 125 mM KCl, 1 mM EGTA, 1 mM KH_2_PO_4_, and 10 mM Tris–MOPS, pH 7.4, at 25°C. Maximum rate of oxygen consumption was recorded for 2 min after addition of 5 mM glutamate & malate, 5 mM succinate, and 0.5 mM ADP. Oligomycin (2.5 µg/ml) was then added to inhibit ATP synthase and block ATP production. The rate of oxygen consumption with oligomycin was recorded for an additional 2 min. The level of proton leak was defined as the ratio of the rate of oxygen consumption in the presence of oligomycin to the rate of ADP-stimulated oxygen consumption (classical *state 3* respiration).

### Adenine nucleotide translocase (ANT) ADP/ATP exchange activity

The ADP/ATP exchange activity of ANT in mitochondria isolated from UCP4-overexpressing and vector cells were determined by inhibitor-stop method with slight modifications [59]. Briefly, mitochondria were isolated according to the above method, and suspended in ice-cold suspension buffer consisting of 125 mM KCl, 1 mM EGTA, and 10 mM Tris–MOPS, pH 7.4. The mitochondria suspension (containing 100 µg mitochondria protein in 500 µl buffer) was incubated with Complex V inhibitor, oligomycin (2.5 µg/ml) for 15 min on ice to stop ATP synthesis before experiments. After this pre-incubation step, 50 nmol of [^14^C]ADP were added to the mitochondrial suspension. The reaction was carried out on ice for 40 sec and immediately stopped by addition of 100 µM atractyloside. The mitochondria were washed five times with fresh suspension buffer containing atractyloside and collected by centrifugation. The resultant mitochondrial pellet was dissolved in 0.1 M NaOH. The radioactivity of [^14^C]ADP in the NaOH solution was determined using a scintillation counter.

### Mitochondrial Complex I activity measurement

Mitochondrial Complex I activity was determined by spectrophotometric enzyme assay as described with slight modifications [60]. Briefly, culture medium of cells growing exponentially was changed two hours before experiment. Cells were washed with PBS and suspended in ice-cooled mitochondrial suspension buffer containing 10 mM Tris-MOPS, 1 mM EGTA, 0.2 M sucrose, pH 7.4, supplemented with protease inhibitor cocktail (Roche). The cellular extracts containing mitochondrial membrane were prepared by homogenization using glass-teflon homogenizer and brief sonication. The resultant lysates were diluted to a concentration of 5 µg/µl. Activity of Complex I was measured by the colorimetric changes after oxidation of NADH at 340 nm wavelength. The mitochondria-rich cellular fraction (200 µg) was pre-incubated in 480 µl reaction buffer containing 35 mM potassium phosphate buffer, pH 7.2, 1 mM EDTA, 1 mg/ml BSA, 2.65 mM KCN, 5 mM MgCl_2_, 2 µg/ml antimycin A, 2.5 mM decylubiquinone for 2 min at 37°C. After the pre-incubation, 20 µl of 5 mM stock β-NADH was added to the reaction mixture, and its absorbance at 340 nm was recorded continuously for 5 min. at every 30 sec. intervals. Samples incubated with 10 µM rotenone (Complex I inhibitor) were used as negative control. The relative Complex I activity was defined as the rate of change of absorbance per unit time of incubation after subtracting the Complex I-insensitive absorbance in the presence of rotenone.

### Mitochondrial Complex II activity measurement

Mitochondrial Complex II activity was determined by standard spectrophotometric enzyme assay as described with slight modifications [60], [61]. The mitochondria-rich cellular fractions were prepared on ice as described above. Activity of Complex II was measured by changes in absorbance at 600 nm after reduction of artificial electron acceptor, 2,6-dichlorophenolindophenol (DCPIP). The cellular fraction (200 µg) was pre-incubated in 480 µl reaction buffer containing 35 mM potassium phosphate buffer, pH 7.2, 1 mM EDTA, 1 mg/ml BSA, 2.65 mM KCN, 50 µM DCPIP, 10 µM rotenone, 5 mM MgCl_2_, 0.2 mM ATP, 10 mM sodium succinate, 2 µg/ml antimycin A for 10 min at 37°C to allow lag period for activation of Complex II. After the pre-incubation, 80 µM decylubiquinone was added to the reaction mixture and its absorbance at 600 nm was recorded continuously for 5 min. at every 30 sec. intervals. Samples incubated with 100 µM 3-nitropropionic acid (3-NP, Complex II inhibitor) were used as negative control. The relative Complex II activity was defined as the rate of change of absorbance per unit time of incubation after subtracting the Complex II-insensitive absorbance readings in the presence of 3-NP.

### Mitochondrial Complex IV activity measurement

Mitochondrial Complex IV activity was measured using a commercial Complex IV human enzyme activity assay kit (Mitosciences) according to manufacturer's procedure. Briefly, cells were washed with PBS, and lysed by brief sonication on ice. Complex IV in the resultant lysates was immune-captured within the wells following overnight incubation at 4°C. After the incubation, the substrate was added to each well to allow reaction to proceed in which the reduced cytochrome *c* was oxidized. The decrease in absorbance at 550 nm was recorded continuously for 2 hours. The relative Complex IV activity was defined as the rate of change of absorbance per unit time of incubation.

### Real-time quantitative RT-PCR and SDS-PAGE western blotting

To determine the steady-state levels of UCP4 and UCP5 mRNAs, total RNA was isolated from UCP4-overexpressing, and vector control cells by Trizol™ reagent and treated with DNase-I to remove genomic DNA. Real-time quantitative RT-PCR was performed using the Taqman EZ RT-PCR reagent kit. The level of β-actin was used to normalize loadings of total RNA. The sequences of primers and probes for UCP4 and UCP5 have been described previously [47].

To determine levels of a number of intracellular proteins, cellular lysates were clarified by centrifugation and the total protein concentrations were measured. SDS-PAGE western blotting was performed using equal amounts of total cellular protein lysate (25 µg), which were electrophoresed on 15% SDS-polyacrylamide gels and then transferred overnight onto PVDF membrane. The resulting blots were blocked with 5% non-fat skimmed milk (Bio-Rad) and probed respectively with various primary antibodies: anti-NDUFS4 (1-E-4) (1∶1000), anti-Complex II (1∶10000), anti-Complex IV subunit II (1∶1000), anti-CoxIV (1∶5000), anti-ANT (1∶1000) and anti-actin (1∶500) antibodies. Resulting blots were incubated with horseradish peroxidase (HRP)-conjugated secondary antibodies (1∶5000) followed by detection using ECL detection reagents. The resultant immunoblots were quantified by computerized scanning densitometry.

### Mitochondrial membrane potential (MMP)

Relative MMP in vector control and UCP4-overexpressing cells were compared by ratiometric measurement of an MMP-sensitive fluorescent dye JC-1. Briefly, cells seeded in 96-well plate (10^5^ cells/well) were stained in culture medium containing 10 µg/ml JC-1 at 37°C in the dark for 15 min. After staining, the medium was replaced by fresh medium. The fluorescent intensity of both red (excitation: 485 nm; emission: 535 nm) and green (excitation: 546 nm; emission: 590 nm) of the stained cells were measured by spectrophotometer (Infinite M200, TECAN). Relative MMP was expressed as the average fluorescent signal of the dye (ratio of red to green signals) from at least six individual wells.

### Co-localization of UCP4 and Complex II - immunocytochemistry

Co-localization of UCP4 and Complex II was demonstrated by co-immunostaining of UCP4 and a 70 kDa Complex II subunit, Fp, in UCP4-overexpressing cells. Briefly, cells were seeded at 50% confluency in chamber slides 24 hours before the immunostaining. Cells were fixed with 4% paraformaldehyde for 30 min at room temperature, and incubated with the diluted goat polyclonal anti-UCP4 primary antibody and mouse monoclonal anti-70 kDa Fp at 4°C overnight, followed by co-incubation with Alexa Fluor 546-conjugated anti-mouse IgG (1∶200) and Alexa Fluor 488-conjugated anti-goat IgG (1∶200) for 1 hour. Images were visualized under confocal microscope (Carl Zeiss LSM 510). Cellular nuclei were counterstained with DAPI.

### Protein-protein interaction between UCP4 and Complex II – co-immunoprecipitation

Mitochondria in UCP4-overexpressing cells were isolated as described above. The resultant mitochondria-enriched pellet was resuspended and lysed by a native lysis buffer under brief sonication (Cell Signaling Ltd.). The mitochondrial lysate (500 µg aliquot) was pre-absorbed by Protein-G-sepharose beads for 30 min. at 4°C to capture non-specific proteins having direct interaction with Protein-G. The pre-cleared supernatant was then incubated with primary antibody against either FLAG or Complex II subunit, Fp, at 4°C overnight with gentle agitation. The protein-antibody complex was then incubated with fresh Protein-G-sepharose beads for 1 hour at 4°C. The resulting antibody-complexes suspension with sepharose beads were washed by Tris-buffer, pH 7.2, supplemented with 0.01% Tween 20, then boiled and denatured in SDS-containing sampling buffer, and run into 12% SDS-polyacrylamide gel. The resultant blots were blocked by skimmed milk and probed with either anti-FLAG or anti-Complex II subunit, Fp. Resulting blots were incubated with HRP-conjugated secondary antibodies followed by detection using ECL detection reagents.

### Cellular ATP levels and the rate and efficiency of ATP synthesis in isolated mitochondria

Both intracellular and mitochondrial ATP levels were assayed using a commercial ATP assay kit (Perkin Elmer). For intracellular ATP level, cells were washed with PBS and harvested under normal culture condition. Total ATP was extracted in lysis buffer containing: 100 mM potassium phosphate buffer (pH 7.8), 2 mM EDTA, 1 mM DTT, and 1% Triton X-100. ATP level in each cell lysate was determined by luciferase bioluminescent assay, in which light emitted was measured by a luminometer.

To compare the efficiency and rate of ATP synthesis between UCP4-overexpressing and vector control cells, fixed amount of isolated mitochondria (100 µg) were incubated in respiratory buffer containing 125 mM KCl, 1 mM EGTA, 1 mM KH_2_PO_4_, 1 mM ADP, and 10 mM Tris–MOPS, pH 7.4, with specific substrates and inhibitors of the respiratory chain complexes. Oxygen consumption was assayed using a Clark-type electrode in microcell as described above. For Complex I-mediated ATP synthesis, 5 mM glutamate and 1 mM malate were added to the mitochondrial suspension to stimulate ATP production. For Complex II-mediated ATP synthesis, 5 mM succinate was added with a Complex I inhibitor, rotenone (2 µM). Mitochondrial suspensions were incubated in a water-jacketed microcell, magnetically stirred at 25°C for 4 min. After the incubation, a specific ATP releasing buffer (Perkin Elmer) was immediately added into the mitochondrial suspension to stop the respiratory chain reactions and to lyse all mitochondria. ATP level was determined in each mitochondrial lysate by luciferase bioluminescent assay. ATP concentrations were determined using a calibration curve of serial ATP dilutions provided with the kit. Basal intra-mitochondrial ATP levels in both UCP4-overexpressing and control mitochondria were determined before incubation. Total mitochondrial protein was used to normalize the amount of ATP produced to allow comparison across groups without confounding influence of differences in mitochondrial loading into the oxygen chamber. Furthermore, changes in oxygen consumption were recorded to monitor the process of substrates-induced ATP synthesis to ensure *state 3* respiration had taken place during the incubation time period. The efficiency of ATP synthesis was defined as (final ATP - initial ATP)/µg mitochondria protein/oxygen atom consumed.

### Statistical analyses

All experiments were repeated in at least four independent treatments. Results were expressed as mean ± S.E.M. Statistical significance was assessed by one-way ANOVA followed by Bonferroni's *post hoc* analysis or by Student's unpaired *t*-test. Differences were considered significant if p<0.05.
